# Bacterial outer-membrane vesicles promote Vγ9Vδ2 T cell oncolytic activity

**DOI:** 10.3389/fimmu.2023.1198996

**Published:** 2023-07-17

**Authors:** Jack Firth, Jingjing Sun, Vaques George, Jian-Dong Huang, Mona Bajaj-Elliott, Kenth Gustafsson

**Affiliations:** ^1^ Department of Biochemical Engineering University College London, London, United Kingdom; ^2^ Chinese Academy of Sciences (CAS) Key Laboratory of Quantitative Engineering Biology, Shenzhen Institute of Synthetic Biology, Shenzhen Institutes of Advanced Technology, Chinese Academy of Sciences, Shenzhen, China; ^3^ School of Biomedical Sciences, Li Ka Shing Faculty of Medicine, The University of Hong Kong, Hong Kong, Hong Kong SAR, China; ^4^ Great Ormond Street Institute of Child Health, University College London (UCL), London, United Kingdom

**Keywords:** OMV, outer-membrane vesicles, immunotherapy, Vγ9Vδ2 T cells, γδ T cells, extracellular vesicles

## Abstract

**Background:**

Increasing evidence suggests the immune activation elicited by bacterial outer-membrane vesicles (OMVs) can initiate a potent anti-tumor immunity, facilitating the recognition and destruction of malignant cells. At present the pathways underlying this response remain poorly understood, though a role for innate-like cells such as γδ T cells has been suggested.

**Methods:**

Peripheral blood mononuclear cells (PBMCs) from healthy donors were co-cultured with *E. coli* MG1655 Δ*pal* Δ*lpxM* OMVs and corresponding immune activation studied by cell marker expression and cytokine production. OMV-activated γδ T cells were co-cultured with cancer cell lines to determine cytotoxicity.

**Results:**

The vesicles induced a broad inflammatory response with γδ T cells observed as the predominant cell type to proliferate post-OMV challenge. Notably, the majority of γδ T cells were of the Vγ9Vδ2 type, known to respond to both bacterial metabolites and stress markers present on tumor cells. We observed robust cytolytic activity of Vγ9Vδ2 T cells against both breast and leukaemia cell lines (SkBr3 and Nalm6 respectively) after OMV-mediated expansion.

**Conclusions:**

Our findings identify for the first time, that OMV-challenge stimulates the expansion of Vγ9Vδ2 T cells which subsequently present anti-tumor capabilities. We propose that OMV-mediated immune activation leverages the anti-microbial/anti-tumor capacity of Vγ9Vδ2 T cells, an axis amenable for improved future therapeutics.

## 1 Introduction

Outer-membrane vesicles (OMVs) are spherical nanoparticles (20-200 nm in diameter), derived from the outer membrane of Gram-negative bacteria. Vesicle blebbing is a homeostatic phenomenon; bacteria utilize OMVs for a wide variety of functions including virulence, nutrient acquisition, and antibiotic resistance (1). OMVs express many microbial-associated molecular patterns (MAMPs) including lipoproteins, lipopolysaccharide (LPS) and peptidoglycan, features that allow the vesicles to elicit a robust immune response (2, 3). In fact, the immunogenicity observed has encouraged significant research into the use of OMVs as vaccines (4). This includes the clinical approval of Bexsero, a meningococcal group B vaccine which contains OMVs derived from *N. meningitidis* (5). More recently, the ability to engineer OMVs has allowed the display of multiple microbial antigens, enabling their adjuvant properties to be leveraged against a range of pathogenic species (6–16).

To improve the clinical translation of an OMV construct, bacterial strains have also been engineered to alleviate challenges with manufacturing and toxicity. Through deletion of the pal scaffold protein (Δ*pal*), bacteria present a hypervesiculating phenotype to significantly increase the production of OMVs (17). In contrast, removal of lipid A acyltransferase (Δ*lpxM*) produces LPS with a penta-acylated structure, exhibiting a reduced affinity for the pattern-recognition receptor TLR4/MD-2 complex (18–20). In doing so, bacteria and OMV Δ*lpxM* mutants induce a less potent and more tolerable immune response (21–24).

Recent evidence indicates that the immunogenic properties of OMVs can also initiate anti-tumor immunity. OMV challenge elicits a sustained oncolytic response against various tumor types in rodents (24, 25). OMVs can not only eradicate an engrafted syngeneic tumor, but also induce the formation of an immunological memory against subsequent challenge (24). Furthermore, tumor neo-antigens expressed on OMVs have been utilized to create a form of cancer vaccine, inducing a potent antibody response in rodents across a variety of cancer types (26–31). Despite major advances, the exact mechanism(s) defining OMV-mediated anti-tumor immunity are yet to be determined.

In the present study, we sought to characterise the response of peripheral blood mononuclear cells (PBMCs) from healthy donors to *E. coli* MG1655 Δ*pal* Δ*lpxM* OMVs. We identify Vγ9Vδ2 T cells as major responders to OMVs with robust oncolytic properties. Targeting the functional characteristics of Vγ9Vδ2 T cells offers additional arsenal for improving future cancer immunotherapies.

## 2 Materials and methods

### 2.1 OMV preparation

OMVs from *E. coli* MG1655 Δ*pal* Δ*lpxM* were isolated as previously described (32), with some modifications. Ultracentrifugation was used to isolate OMVs *via* pelleting at 235,000 x g for 2 hours at 8°C using a fixed angle 45 Ti rotor (Beckman Coulter). Pellets were washed *via* resuspension in PBS and pelleting again, before final resuspension in PBS.

### 2.2 OMV characterization

The size and concentration of OMVs was measured by nanoparticle tracking using a NanoSight NS300 (Malvern Panalytical) and analysed by NanoSight NTA software (Malvern Panalytical). Where possible samples were diluted in PBS to obtain between 20 and 80 particles per frame. Results consisted of five measurements each using 60 second recordings. Camera sensitivity, gain and detection threshold were set to 16, 10 and 4 respectively, whilst samples were administered and recorded under controlled flow using the NanoSight syringe pump and script control system.

### 2.3 PBMC stimulation with *Escherichia coli* MG1655 Δ*pal* Δ*lpxM* OMVs

Whole blood from six healthy donors (HD1-HD6) stored in sodium citrate was purchased from Cambridge Bioscience and delivered<24 hours after sampling. Upon receipt, PBMCs were immediately isolated *via* ficol density gradient separation. Samples were then washed in supplemented RPMI 1640 media and PBS, before being resuspended in media for counting (NC3000 nucleocounter, Chemometec). All supplemented RPMI 1640 (Life Technologies) contained, 10% heat-inactivated foetal bovine serum (FBS) (One Shot, Gibco) and 2 mM L-glutamine (Gibco).

PBMCs were cultured in supplemented pre-warmed RPMI 1640 media at a density of 1x10^6^/ml. Cells were dosed with 100 μl of *E. coli* MG1655 Δ*pal* Δ*lpxM* OMVs at a concentration of 1x10^9^/ml or 1x10^10^/ml to give a ratio of 1x10^3^:1 or 1x10^4^:1 (OMVs : PBMCs) respectively. Samples were incubated for either 24 hours or 5 days at 37°C and 5% CO_2_, after which cells were pelleted for cell surface marker analysis by flow cytometry, and the supernatant used for cytokine analysis.

Quantification of cytokine production was determined by ELISA. IFN-γ analysis used an IFN gamma Human Uncoated ELISA Kit (ThermoFisher Scientific). Granzyme B was measured *via* an Ella Automated Immunoassay System (Ella, Protein Simple) using a 16 x 4 Custom Simple Plex Assay Panel, performed as per the manufacturer’s instructions. A custom ELISA array kit (Multi-Analyte ELISArray, Qiagen) was also used to screen a panel of cytokines and chemokines. For the ELISArray, cytokine concentration was measured *via* absorbance at OD_450nm_ normalised to a positive control sample as directed by the protocol provided.

### 2.4 γδ T cell activation with *Escherichia coli* MG1655 Δ*pal* Δ*lpxM* OMVs

1ml of PBMCs (1x10^6^/ml) from three healthy donors were incubated for 10 days with either 2x10^10^ *E. coli* MG1655 Δ*pal* Δ*lpxM* OMVs (20000:1 ratio), 5 μM zoledronate (zoledronic acid monohydrate, Sigma-Aldrich) or PBS. In all cases, cells were also supplemented with 100 IU/ml IL-2 (human IL-2 IS premium grade, Miltenyi Biotec), which continued every three days along with re-adjustment of media. At day 3, 500 μl media was added to each well to increase the usable volume. Upon completion of each incubation, PBMCs in each sample were stained for various markers and analysed by flow cytometry (**Supplementary Table 1**).

### 2.5 Flow cytometry

Samples were washed twice in eBioscience flow cytometry staining buffer (Invitrogen) and incubated in Fc-block (Human TruStain FcX, Biolegend) for 15 minutes at room temperature. After pelleting, cells were then stained for surface markers using antibodies listed in **Supplementary Table 1**. Live cells were identified using LIVE/DEAD Fixable Aqua Dead Cell Stain (Life Technologies). After staining, cells were washed in staining buffer and fixed in Cytofix buffer (BD Biosciences) for 30 minutes (4°C) before analysis. Controls included unstained cells, as well as antibodies affixed to UltraComp eBeads Compensation Beads (Invitrogen) for compensation controls. Cells from each experiment were stained with isotype controls of each antibody, and stained heat killed cells (56°C, 15 mins) combined with live cells (1:1) were used as a live/dead control. Fluorescence minus one (FMO) controls were used to appropriately gate cell populations.

### 2.6 γδ T cell isolation

γδ T cells were isolated from activated PBMCs using EasySep Human Gamma/Delta T Cell Isolation Kit (StemCell Technologies), according to the manufacturer’s instructions. The isolated supernatant was then resuspended in supplemented RPMI1640 media before use. To confirm isolation purity, cells were analysed by flow cytometry and γδ T cells identified as CD3^+^ αβTCR^-^ (**Supplementary Table 1**). This form of identification has been shown to accurately determine γδ T cells and was validated in this study as matching the cell proportion when gating with CD3^+^ Vδ1^+^ + CD3^+^ Vδ2^+^ cells (**Supplementary Figure 1**). Isolated cells were confirmed as >90% purity before use in further experiments (**Supplementary Figure 1**).

### 2.7 γδ T cell-mediated Nalm6 cell killing

To determine the killing capacity of γδ T cells against Nalm6 cells, 2x10^5^ Nalm6 cells (courtesy of Qasim Rafiq, Department of Biochemical Engineering, UCL) were incubated with either 2x10^5^ or 6x10^5^ of zoledronate or OMV-activated γδ T cells. To ensure a sufficient cell number, PBMCs were activated for 14-days before isolation of γδ T cells, following the protocol described in section 2.4. To identify Nalm6 cell killing, cells from each sample were stained for expression of CD3, with Nalm6 cells identified as CD3^+^ αβTCR^+^.

### 2.8 γδ T cell-mediated SkBr3 cell killing

The killing of SkBr3 cells (HTB-30, ATCC) was explored using the MTS assay protocol described previously by Tokuyama et al. (33). SkBr3 cells, cultured in DMEM Glutamax + 5% FBS, were seeded overnight at 37°C and 5% CO_2_ in a 96-well plate at a density of 1x10^4^ cells per well. The media was then replaced with supplemented RPMI 1640 containing 14-day activated and isolated γδ T cells (with either zoledronate or OMV stimulation) at various effector to target (E:T) ratios, with media alone added as a negative control. Wells containing γδ T cells alone were used as γδ T cell controls, whilst wells with media alone were used as a blank. After 18 hours, media containing the nonadherent γδ T cells was removed and replaced with fresh media containing the MTS reagent (R&D systems). After a 3-hour incubation, optical density was measured at 490 nm and % cytotoxicity calculated as follows:


$$1-\frac{{\text{OD}}_{490\text{nm}}\text{ SkBr}3\;\&\text{ γδT cells}-{\text{OD}}_{490\text{nm}}\text{ γδT cell control}}{{\text{OD}}_{490\text{nm}}\text{ SkBr}3\text{ control}-{\text{OD}}_{490\text{nm}}\text{ media blank}}$$

## 3 Results

### 3.1 *Escherichia coli* MG1655 Δ*pal* Δ*lpxM* OMVs promote an inflammatory immune response

OMV immunotherapy seeks to leverage local immune-activation to facilitate tumor-cell recognition and lysis. It was therefore important to first characterise the OMV-mediated host immune response. This analysis was particularly necessary to ensure the inflammatory response was not completely abolished given the use of an OMV construct with reduced immunogenic properties.

**Figure 1:**
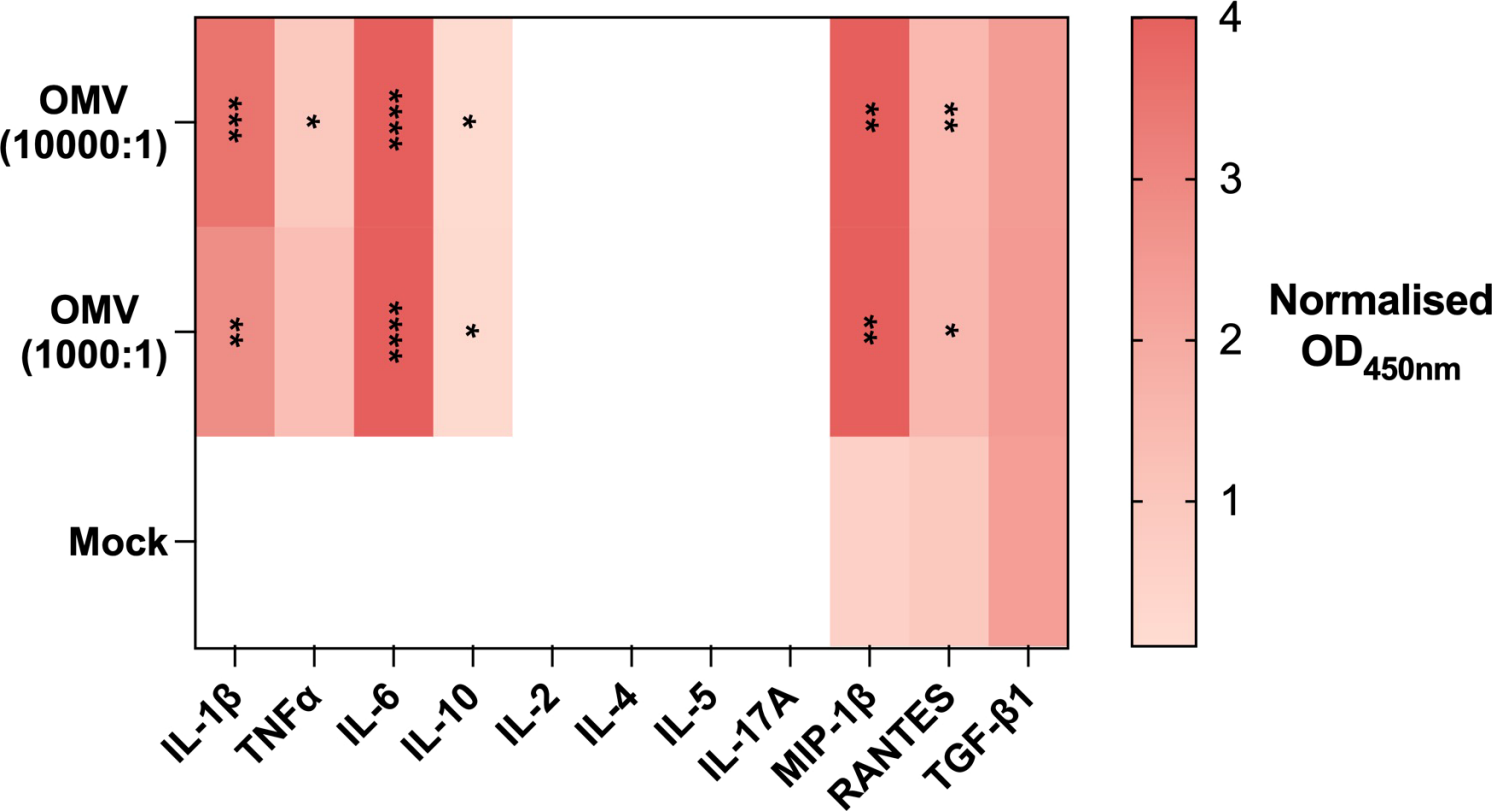
*E. coli* MG1655 Δ*pal* Δ*lpxM* OMVs induce an inflammatory response. Heatmap of cytokines released by PBMC in response to stimulation with PBS (Mock), *E. coli* MG1655 Δ*pal* Δ*lpxM* OMVs at 1x10^4^ vesicles per cell (10000:1), and 1x10^3^ vesicles per cell (1000:1). Cytokine concentration measured *via* absorbance at OD_450nm_. Blank space indicates absence of detectable cytokine. Data displayed as the mean of three replicates. ****P<0.0001,***P<0.001, **P<0.01, *P< 0.05, analysed by one-way ANOVA with Tukey’s post-test. The P-value displayed indicates significance between the respective OMV arm and Mock control.

Despite the attenuated LPS provided by Δ*lpxM*, *E. coli* MG1655 Δ*pal* Δ*lpxM* OMVs stimulated PBMC to generate a robust immune response after 24 hrs. The immunogenic nature of the response was characterised by the release of pro-inflammatory cytokines IL-1β, TNFα and IL-6, as well as the anti-inflammatory cytokine IL-10 (**Figure 1**). We also observed the production of lymphocyte-recruiting chemokines, RANTES (CCL5) and MIP-1β (CCL4).

Notably, the cytokine release profile did not indicate the presence of cytokines associated with lymphocyte activation including IL-2, IL-4, IL-5, and IL-17A. There was also no discernible effect on the release of the myeloid chemoattractant TGF-β1.

### 3.2 *Escherichia coli* MG1655 Δ*pal* Δ*lpxM* OMVs activate cytotoxic lymphocytes

Since lymphocytes produce IL-2 once activated, it is possible that at 24 hours the quantity produced was not sufficient for detection. The incubation period was therefore extended to 5 days, with analysis focused on individual lymphocyte markers to determine cell-specific activation.

**Figure 2:**
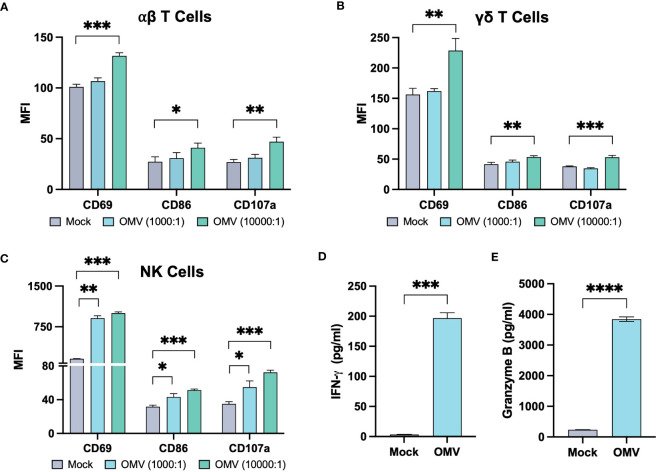
*E*. *coli* MG1655 Δ*pal* Δ*lpxM* OMVs activate αβ T cells, γδ T cells, and NK cells. Activation marker expression on αβ T cells **(A)**, γδ T cells **(B)** and NK cells **(C)**, five days after stimulation of PBMCs with PBS (Mock), *E. coli* MG1655 Δ*pal* Δ*lpxM* OMVs at 1x10^4^ vesicles per cell (10000:1), and 1x10^3^ vesicles per cell (1000:1). Data is presented from one donor and is representative of three donors (**Supplementary Figures 2**, **3**). Expression measured using median fluorescence intensity (MFI). Release of IFN-γ **(D)** and granzyme B **(E)** by PBMC, five days after stimulation with *E. coli* MG1655 Δ*pal* Δ*lpxM* OMVs (OMV) at 1x10^4^ vesicles per cell, or PBS (Mock). Data is presented from one donor and is representative of three donors (**Supplementary Figure 6**). Data displayed as the mean ± SD from a representative experiment (n=3). ****P<0.0001, ***P<0.001, **P<0.01, *P< 0.05, analysed by one-way ANOVA with Tukey’s post-test **(A-C)**, and by unpaired Welsch’s t-test **(D, E)**.

*E. coli* MG1655 Δ*pal* Δ*lpxM* OMVs induced a broad expression of markers CD69, CD86 and CD107a across αβ T cells, γδ T cells and NK cells (**Figures 2A-C**; **Supplementary Figures 2**, **3**). Activation marker expression as a proportion of cells expressing the marker is presented in **Supplementary Figures 4, 5**. The effect appeared to be concentration dependant, with a low concentration of OMVs only inducing activation marker expression in NK cells. Most notable however was the expression of CD107a, as this marker is present within lytic vesicles and displayed on the cell surface upon degranulation. Expression therefore suggests the cells were actively releasing cytotoxic factors; the release of granzyme B as well as IFN-γ was confirmed on further analysis (**Figures 2D, E**; **Supplementary Figure 6**).

Overall, activation markers and cytokines induced by OMVs suggest that the vesicles can induce an inflammatory milieu which may not only recruit lymphocytes, but additionally activate the cells to express a cytotoxic phenotype.

### 3.3 Vγ9Vδ2 T cells are the primary lymphocyte to proliferate in response to *Escherichia coli* MG1655 Δ*pal* Δ*lpxM* OMVs

To investigate the effect of OMV-stimulation on the expansion of cytotoxic lymphocytes, PBMCs were stimulated with either OMVs or zoledronate. Of particular interest was the expansion of γδ T cells and NK cells, as their MHC-independent activation mechanisms provide an ability to recognize both bacterial and malignant antigens simultaneously.

In line with the widespread activation of all lymphocytes tested, there was a dramatic expansion of the total immune cell population in response to OMVs. The PBMCs were seen to reach a similar total concentration across all conditions (**Supplementary Figure 7**), potentially due to limited resources inhibiting further growth.

**Figure 3:**
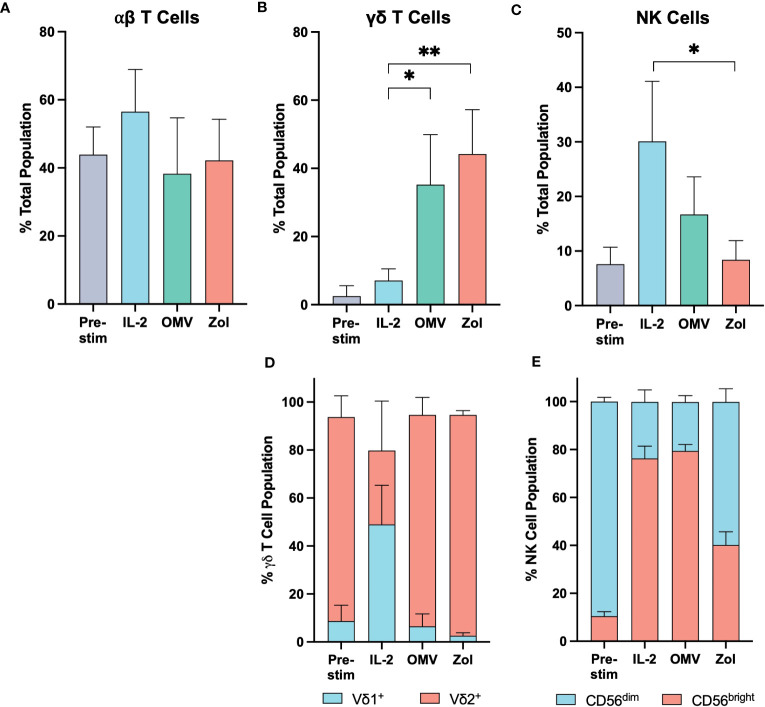
Vγ9Vδ2 T cells proliferate significantly in response to *E*. *coli* MG1655 Δ*pal* Δ*lpxM* OMVs. The relative proportion of αβ T cells **(A)**, γδ T cells **(B)**, and NK cells **(C)** within the PBMC population before stimulation (Pre-stim), and after a 10-day expansion period stimulated with IL-2 alone (IL-2), OMVs with IL-2 (OMV), and zoledronate with IL-2 (Zol). **(D)** Relative proportion of Vδ1^+^ and Vδ2^+^ sub-types within the γδ T cell population. **(E)** Relative proportion of CD56^bright^ and CD56^dim^ sub-types within the NK cell population. Data are presented as the mean ± SD (n=3). **P<0.01, *P< 0.05, analysed by one-way ANOVA with Tukey’s post-test comparing each activation stimuli to the other.

The proportion of αβ T cells remained constant upon OMV activation, relative to the pre-stimulated control (**Figure 3A**; **Supplementary Figure 8**). In contrast, OMV + IL-2 activation induced γδ T cells to expand to around 35% of the total cell population, significantly greater than the ~7% achieved with IL-2 alone (**Figure 3B**). This expansion was similar to that observed in response to zoledronate + IL-2, a compound utilized for the specific expansion of the Vγ9Vδ2 (Vδ2^+^) subtype of γδ T cells. Whilst it is interesting to note that IL-2 alone facilitated the growth of the Vδ1^+^ subtype within the γδ T cell population, we observed a dominance in the proportion of Vγ9Vδ2 T cells in response to both zoledronate and OMV activation (**Figure 3D**).

Since the overall cell number was similar to that seen with IL-2 alone, it appears that the proliferation of Vγ9Vδ2 T cells in response to OMVs was mostly at the expense of NK cell expansion (**Figure 3C**). Their relative proportion in the experimental cell milieu was not significantly different compared to the starting population, though there was an apparent phenotypic shift to the immunoregulatory CD56^bright^ subtype. Given their similarity in proportion however, it is likely that this preference to CD56^bright^ cells was driven at least in part through the effect of IL-2 supplementation (**Figure 3E**).

### 3.4 OMV-activated γδ T cells retain their tumor-killing capabilities

**Figure 4:**
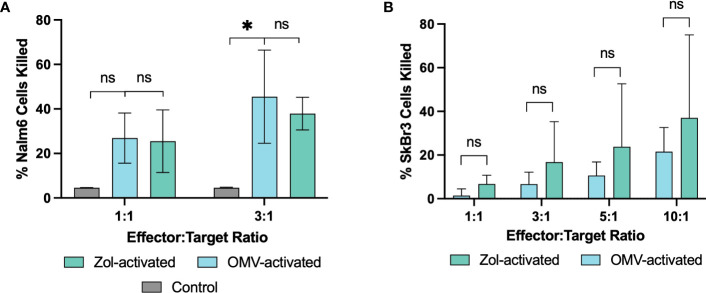
Vγ9Vδ2 T cells retain their oncolytic functionality after activation with *E*. *coli* MG1655 Δ*pal* Δ*lpxM* OMVs. Oncolytic capacity of γδ T cells after activation with OMVs or zoledronate against Nalm6 **(A)**, and SkBr3 **(B)** cancer cell lines. Background cell death of Nalm6 cells measured using media without the addition of effector cells (Control), whilst SkBr3 killing is determined as a relative proportion of background cell death. Data are presented as the mean ± SD (n=3). *P< 0.05, ns = non-significant, analysed by one-way ANOVA with Tukey’s post-test **(A)** or by paired Welsch’s t-test **(B)**.

Though it was apparent that γδ T cells respond to OMVs, it was necessary to confirm their potential oncolytic activity despite the microbial means of activation. Isolated γδ T cells were expanded with either OMVs or zoledronate, and their killing capacity determined against a leukaemic (Nalm6) and breast cancer (SkBr3) cell line. Indeed, OMV-activated γδ T cells were able to effectively initiate cell killing (**Figure 4**). Whilst inter-donor variability meant Nalm6 killing at a 1:1 effector to target (E:T) ratio did not reach statistical significance, the oncolytic capacity of OMV-expanded cells was equivalent to that of cells activated with zoledronate. This killing effect was also observed in SkBr3, again not significantly different to that of cells activated with zoledronate (**Figure 4**).

## 4 Discussion

As highly immunogenic bacterial nanoparticles, OMVs can encourage the recognition and destruction of malignant cells by the host immune system (24, 25). Immunotherapeutic development of OMVs has fostered a distinct need to further characterise the underlying immune response generated. Herein, we demonstrated that Vγ9Vδ2 T cells are the primary lymphocytes that respond to prolonged OMV exposure and retain the capability to kill tumor cells post-activation. To facilitate future clinical use, we leveraged Δ*pal* and Δ*lpxM* deletions to both improve vesicle production (by increasing outer membrane flexibility) and reduce toxicity (through the inactivation of lipid A).

Despite the attenuated toxicity afforded by Δ*lpxM* mutation, OMVs fostered a robust inflammatory response with the potential to recruit further lymphocytes to the site of activation. It is possible that this immunogenic activation elicited by OMVs could itself facilitate a therapeutic response. Exogenous induction of acute inflammation has been proposed as means to re-structure the immunological phenotype of the tumor microenvironment, fostering an oncolytic response based around the polarisation of macrophages to the anti-tumor, M1 phenotype (34). Interestingly, the inflammatory factors observed in this research (e.g. IL-1β, TNFα, RANTES, and MIP-1β) are primarily associated with monocytes, including M1 macrophages, after stimulation with both OMVs and bacteria (35–37).

Given their direct involvement in surveillance and killing, the activation of cytotoxic lymphocytes is critical for an effective anti-tumor response. Expression of markers CD69 and CD86 were indicative of such activation across αβ T cells, γδ T cells and NK cells in response to OMVs (38–40). Moreover, the vesicles elicited a cytotoxic phenotype, characterised by degranulation and the release of cytolytic factors granzyme B and IFN-γ. The release of IFN-γ is particularly important in an immunotherapeutic context given its role in Th1 differentiation, inducing apoptosis, and upregulating cell-death inducing ligands (e.g. Fas-L and TRAIL) (41–44).

The expansion of specific lymphocyte populations can dramatically change the composition of the immune environment, and as a result the ability to instigate an effective anti-tumor immune response. Of particular interest was the role of both γδ T cell and NK cells, as they leverage MHC-independent activation mechanisms and therefore are not restricted to microbial targets upon activation with OMVs. Indeed, our study indicates that γδ T cells form a significant proportion of the local immune microenvironment in response to OMV stimulation.

γδ T cells are unique in their ability to recognize antigens in an HLA-unrestricted manner, responding to broad markers of microbial presence and endogenous cell stress through both the γδ T cell receptor as well as various cytotoxicity and NK cell receptors (e.g. NKG2D) (45–49). The Vγ9Vδ2 subtype recognizes both microbial antigens as well as metabolites of the mevalonate biosynthetic pathway (e.g. isopentenyl pyrophosphate (IPP)) (47, 50, 51). This dual activation allows OMVs to respond to microbial antigens whilst retaining the ability to kill, providing a direct mechanism by which OMV challenge induces anti-tumor immunity. In fact, Vγ9Vδ2 T cells possess a variety of unique characteristics that make them ideally poised for leveraging as a cancer immunotherapy tool; features including, broad antigen recognition, antibody-dependent cellular cytotoxicity (ADCC), and professional antigen presenting capabilities (33, 52–55).

Whilst the exact mechanisms of OMV-activation require deeper investigation, it is hypothesised that the metabolite HMB-PP (an intermediate of the methylerythritol 4-phosphate pathway) present in *E. coli* can stimulate γδ T cells. This can occur both directly (via BTN3A/CD277 interaction) and indirectly through the accumulation of endogenous pyrophosphates (e.g. IPP) in surrounding immune cells (47, 51, 56, 57). TLR4 receptor is also expressed on γδ T cells and can be further modulated in response to bacterial antigen presentation *via* dendritic cells (58, 59). Since OMVs display a large array of TLR agonists, a direct mechanism of OMV-mediated γδ T cell activation is also likely, though this hypothesis warrants further investigation. Given the direct and indirect pathways that govern γδ T cell activation, many of which are yet to be fully understood, identifying the specific mechanism of OMV-mediated stimulation will require significant research beyond the scope of this paper. Despite this, a full appreciation could allow for a more precise manipulation of the anti-tumor response.

It must also be considered that the deliberate induction of an acute inflammatory response by OMVs may lead to adverse toxicity. IL-6 and TNFα have both been implicated as playing significant roles in the development of a cytokine storm, suggesting a risk of such events in response to OMV-stimulation (60). However the unique characteristics of OMVs may mitigate such toxicity. The broad engagement of innate receptors enables a concurrent induction of regulatory pathways, evidenced by the release of IL-10, which can suppress the release of IL-6 and TNFα particularly from monocytes (61). Furthermore, the use of an Δ*lpxM* mutant strain expressing penta-acylated LPS can alleviate the toxicity risk through its reduced immunogenic properties. Indeed, OMVs derived from a similar strain failed to induce any observable toxicity in rodents (24).

Importantly though, the poor recapitulation of CRS in rodent models makes judgement of the OMVs safety profile complicated. Rodent studies may therefore overstate the reduced immunogenic benefit of penta-acylated LPS. It is also unclear that the IL-10 released can successfully limit adverse immune responses and may even facilitate tumor progression by suppressing anti-tumor macrophages within the TME. Consequently, understanding the toxicity profile of OMVs should be prioritised when it comes to future development of the concept.

Similar to the use of an Δ*lpxM* mutant to provide an improved toxicity profile, it is also possible to tailor the OMV immune response to improve its therapeutic effect. In contrast to the use of mammalian extracellular vesicles (EVs) (62), tumor antigen expression on OMVs offers a means to leverage the OMV backbone as an antigen-adjuvant vehicle, thus providing robust and sustained anti-tumor immunity (26–31). Given the expansion of γδ T cells in response to OMVs, their professional antigen presenting capacity may also be exploited to facilitate the cross-presentation of displayed antigens. In fact, functionalisation of OMVs through the expression of various proteins may also serve to modulate the γδ T cell response, evidenced through the presentation of checkpoint inhibitors on both mammalian and bacterial EVs (63, 64). Overall, our findings support the hypothesis that Vγ9Vδ2 T cells are a crucial component of the OMV anti-tumor immune response, providing new opportunities to design more effective OMV-mediated immunotherapies.

## Data availability statement

The raw data supporting the conclusions of this article will be made available by the authors, without undue reservation.

## Author contributions

JF and KG contributed to the conception and design of the study. JF performed the experiments, data analysis and prepared the manuscript. JS and JH developed the bacterial strain used. JS, JH, VG and MB-E provided additional scientific support. MB-E and KG reviewed and edited the manuscript. All authors contributed to the article and approved the submitted version.

## References

[1] Toyofuku M Schild S Kaparakis-Liaskos M Eberl L. Composition and functions of bacterial membrane vesicles. Nat Rev Microbiol (2023) 21:415–30. doi: 10.1038/s41579-023-00875-5 36932221

[2] Cecil JD O’Brien-Simpson NM Lenzo JC Holden JA Singleton W Perez-Gonzalez A. Outer membrane vesicles prime and activate macrophage inflammasomes and cytokine secretion *In vitro* and *In vivo* . Front Immunol (2017) 8:1017. doi: 10.3389/fimmu.2017.01017 28890719 PMC5574916

[3] Bielaszewska M Marejkova M Bauwens A Kunsmann-Prokscha L Mellmann A Karch H. Enterohemorrhagic escherichia coli O157 outer membrane vesicles induce interleukin 8 production in human intestinal epithelial cells by signaling *via* toll-like receptors TLR4 and TLR5 and activation of the nuclear factor NF-kappaB. Int J Med Microbiol (2018) 308:882–9. doi: 10.1016/j.ijmm.2018.06.004 29934223

[4] Acevedo R Fernández S Zayas C Acosta A Sarmiento ME Ferro VA. Bacterial outer membrane vesicles and vaccine applications. Front Immunol (2014) 5:121. doi: 10.3389/fimmu.2014.00121 24715891 PMC3970029

[5] Bai X Findlow J Borrow R. Recombinant protein meningococcal serogroup b vaccine combined with outer membrane vesicles. Expert Opin Biol Ther (2011) 11:969–85. doi: 10.1517/14712598.2011.585965 21615224

[6] Schroeder J Aebischer T. Recombinant outer membrane vesicles to augment antigen-specific live vaccine responses. Vaccine (2009) 27:6748–54. doi: 10.1016/j.vaccine.2009.08.106 19748581

[7] Fantappie L de Santis M Chiarot E Carboni F Bensi G Jousson O. Antibody-mediated immunity induced by engineered escherichia coli OMVs carrying heterologous antigens in their lumen. J Extracell Vesicles (2014) 3:24015. doi: 10.3402/jev.v3.24015 PMC4131003 25147647

[8] Kuipers K Daleke-Schermerhorn MH Jong WSP ten Hagen-Jongman CM van Opzeeland F Simonetti E. Salmonella outer membrane vesicles displaying high densities of pneumococcal antigen at the surface offer protection against colonization. Vaccine (2015) 33:2022–9. doi: 10.1016/j.vaccine.2015.03.010 25776921

[9] Huang W Wang S Yao Y Xia Y Yang X Li K. Employing escherichia coli-derived outer membrane vesicles as an antigen delivery platform elicits protective immunity against acinetobacter baumannii infection. Sci Rep (2016) 6:37242. doi: 10.1038/srep37242 27849050 PMC5110958

[10] Rappazzo CG Watkins HC Guarino CM Chau A Lopez JL DeLisa MP. Recombinant M2e outer membrane vesicle vaccines protect against lethal influenza a challenge in BALB/c mice. Vaccine (2016) 34:1252–8. doi: 10.1016/j.vaccine.2016.01.028 26827663

[11] van den Berg van Saparoea HB Houben D Kuijl C Luirink J Jong WSP. Combining protein ligation systems to expand the functionality of semi-synthetic outer membrane vesicle nanoparticles. Front Microbiol (2020) 11:890. doi: 10.3389/fmicb.2020.00890 32477305 PMC7235339

[12] König E Gagliardi A Riedmiller I Andretta C Tomasi M Irene C. Multi-antigen outer membrane vesicle engineering to develop polyvalent vaccines: the staphylococcus aureus case. Front Immunol (2021) 12:752168. doi: 10.3389/fimmu.2021.752168 34819933 PMC8606680

[13] Klouwens MJ Salverda MLM Trentelman JJ Ersoz JI Wagemakers A Gerritzen MJH. Vaccination with meningococcal outer membrane vesicles carrying borrelia OspA protects against experimental Lyme borreliosis. Vaccine (2021) 39:2561–7. doi: 10.1016/j.vaccine.2021.03.059 33812741

[14] Thapa HB Müller AM Camilli A Schild S. An intranasal vaccine based on outer membrane vesicles against SARS-CoV-2. Front Microbiol (2021) 12:752739. doi: 10.3389/fmicb.2021.752739 34803974 PMC8602898

[15] van der Ley PA Zariri A van Riet E Oosterhoff D Kruiswijk CP. An intranasal OMV-based vaccine induces high mucosal and systemic protecting immunity against a SARS-CoV-2 infection. Front Immunol (2021) 12:781280. doi: 10.3389/fimmu.2021.781280 34987509 PMC8721663

[16] Jiang L Driedonks TAP Jong WSP Dhakal S Bart van den Berg van Saparoea H Sitaras I. A bacterial extracellular vesicle-based intranasal vaccine against SARS-CoV-2 protects against disease and elicits neutralizing antibodies to wild-type and delta variants. J Extracell Vesicle (2022) 11:e12192. doi: 10.1002/jev2.12192 PMC8920961 35289114

[17] Deatherage BL Lara JC Bergsbaken T Barrett SLR Lara S Cookson BT. Biogenesis of bacterial membrane vesicles. Mol Microbiol (2009) 72:1395–407. doi: 10.1111/j.1365-2958.2009.06731.x PMC2745257 19432795

[18] Coats SR Pham T-TT Bainbridge BW Reife RA Darveau RP. MD-2 mediates the ability of tetra-acylated and penta-acylated lipopolysaccharides to antagonize *Escherichia coli* lipopolysaccharide at the TLR4 signaling complex. J Immunol (2005) 175:4490–8. doi: 10.4049/jimmunol.175.7.4490 16177092

[19] Teghanemt A Zhang D Levis EN Weiss JP Gioannini TL. Molecular basis of reduced potency of underacylated endotoxins. J Immunol (2005) 175:4669–76. doi: 10.4049/jimmunol.175.7.4669 16177114

[20] Zimmer SM Zughaier SM Tzeng Y-L Stephens DS. Human MD-2 discrimination of meningococcal lipid a structures and activation of TLR4. Glycobiology (2007) 17:847–56. doi: 10.1093/glycob/cwm057 17545685

[21] Somerville JE Cassiano L Bainbridge B Cunningham MD Darveau RP. A novel escherichia coli lipid a mutant that produces an antiinflammatory lipopolysaccharide. J Clin Invest (1996) 97:359–65. doi: 10.1172/JCI118423 PMC507025 8567955

[22] van der Ley P Steeghs L Hamstra HJ ten Hove J Zomer B van Alphen L. Modification of lipid a biosynthesis in neisseria meningitidis lpxL mutants: influence on lipopolysaccharide structure, toxicity, and adjuvant activity. Infect Immun (2001) 69:5981–90. doi: 10.1128/IAI.69.10.5981-5990.2001 PMC98725 11553534

[23] Ranallo RT Kaminski RW George T Kordis AA Chen Q Szabo K. Virulence, inflammatory potential, and adaptive immunity induced by shigella flexneri msbB mutants. Infection Immun (2010) 78:400–12. doi: 10.1128/IAI.00533-09 PMC2798193 19884336

[24] Kim OY Park HT Dinh NTH Choi SJ Lee J Kim JH. Bacterial outer membrane vesicles suppress tumor by interferon-gamma-mediated antitumor response. Nat Comms (2017) 8:626. doi: 10.1038/s41467-017-00729-8 PMC5606984 28931823

[25] Aly RGO El-Enbaawy MIH Abd El-Rahman SS Ata NS. Antineoplastic activity of salmonella typhimurium outer membrane nanovesicles. Exp Cell Res (2021) 399:112423. doi: 10.1016/j.yexcr.2020.112423 33338480

[26] Grandi A Tomasi M Zanella I Ganfini L Caproni E Fantappiè L. Synergistic protective activity of tumor-specific epitopes engineered in bacterial outer membrane vesicles. Front Oncol (2017) 7. doi: 10.3389/fonc.2017.00253 PMC5681935 29164053

[27] Wang S Huang W Li K Yao Y Yang X Bai H. Engineered outer membrane vesicle is potent to elicit HPV16E7-specific cellular immunity in a mouse model of TC-1 graft tumor. IJN (2017) 12:6813–25. doi: 10.2147/IJN.S143264 PMC5602458 28979120

[28] Grandi A Fantappiè L Irene C Valensin S Tomasi M Stupia S. Vaccination with a FAT1-derived b cell epitope combined with tumor-specific b and T cell epitopes elicits additive protection in cancer mouse models. Front Oncol (2018) 8. doi: 10.3389/fonc.2018.00481 PMC6212586 30416985

[29] Cheng K Zhao R Li Y Qi Y Wang Y Zhang Y. Bioengineered bacteria-derived outer membrane vesicles as a versatile antigen display platform for tumor vaccination *via* plug-and-Display technology. Nat Commun (2021) 12:2041. doi: 10.1038/s41467-021-22308-8 33824314 PMC8024398

[30] Li Y Ma X Yue Y Zhang K Cheng K Feng Q. Rapid surface display of mRNA antigens by bacteria-derived outer membrane vesicles for a personalized tumor vaccine. Adv. Materials (2022) 34:2109984. doi: 10.1002/adma.202109984 35315546

[31] Zhuang W-R Wang Y Nie W Lei Y Liang C He J. Bacterial outer membrane vesicle based versatile nanosystem boosts the efferocytosis blockade triggered tumor-specific immunity. Nat Commun (2023) 14:1675. doi: 10.1038/s41467-023-37369-0 36966130 PMC10039929

[32] Chutkan H Macdonald I Manning A Kuehn MJ. Quantitative and qualitative preparations of bacterial outer membrane vesicles. Methods Mol Biol (2013) 966:259–72. doi: 10.1007/978-1-62703-245-2_16 PMC4317262 23299740

[33] Tokuyama H Hagi T Mattarollo SR Morley J Wang Q Fai-So H. Vγ9Vδ2 T cell cytotoxicity against tumor cells is enhanced by monoclonal antibody drugs–rituximab and trastuzumab. Int J Cancer (2008) 122:2526–34. doi: 10.1002/ijc.23365 18307255

[34] Zhao H Wu L Yan G Chen Y Zhou M Wu Y. Inflammation and tumor progression: signaling pathways and targeted intervention. Sig Transduct Target Ther (2021) 6:263. doi: 10.1038/s41392-021-00658-5 PMC8273155 34248142

[35] Tavano R Franzoso S Cecchini P Cartocci E Oriente F Aricò B. The membrane expression of neisseria meningitidis adhesin a (NadA) increases the proimmune effects of MenB OMVs on human macrophages, compared with NadA- OMVs, without further stimulating their proinflammatory activity on circulating monocytes. J Leukoc. Biol (2009) 86:143–53. doi: 10.1189/jlb.0109030 19401383

[36] Arango Duque G Descoteaux A. Macrophage cytokines: involvement in immunity and infectious diseases. Front Immunol (2014) 5:1–12. doi: 10.3389/fimmu.2014.00491 PMC4188125 25339958

[37] Vanaja SK Russo AJ Behl B Banerjee I Yankova M Deshmukh SD. Bacterial outer membrane vesicles mediate cytosolic localization of LPS and caspase-11 activation. Cell (2016) 165:1106–19. doi: 10.1016/j.cell.2016.04.015 PMC4874922 27156449

[38] Santis AG López-Cabrera M Sánchez-Madrid F Proudfoot N. Expression of the early lymphocyte activation antigen CD69, a c-type lectin, is regulated by mRNA degradation associated with AU-rich sequence motifs. Eur J Immunol (1995) 25:2142–6. doi: 10.1002/eji.1830250804 7664776

[39] Hakamada-Taguchi R Kato T Ushijima H Murakami M Uede T Nariuchi H. Expression and co-stimulatory function of B7-2 on murine CD4+ T cells. Eur J Immunol (1998) 28:865–73. doi: 10.1002/(SICI)1521-4141(199803)28:03<865::AID-IMMU865>3.0.CO;2-T 9541581

[40] Jeannin P Herbault N Delneste Y Magistrelli G Lecoanet-Henchoz S Caron G. Human effector memory T cells express CD86: a functional role in naive T cell priming. J Immunol (1999) 162:2044–8. doi: 10.4049/jimmunol.162.4.2044 9973476

[41] Xu X Fu X-Y Plate J Chong AS-F. IFN-y induces cell growth inhibition by fas-mediated apoptosis: requirement of STATI protein for up-regulation of fas and FasL expression. Cancer Res. (1998) 58:2832–7. 9661898

[42] Liu F Hu X Zimmerman M Waller JL Wu P Hayes-Jordan A. TNFα cooperates with IFN-γ to repress bcl-xL expression to sensitize metastatic colon carcinoma cells to TRAIL-mediated apoptosis. PloS One (2011) 6:e16241. doi: 10.1371/journal.pone.0016241 21264227 PMC3022032

[43] Corthay A Skovseth DK Lundin KU Røsjø E Omholt H Hofgaard PO. Primary antitumor immune response mediated by CD4+ T cells. Immunity (2005) 3:371–83. doi: 10.1016/j.immuni.2005.02.003 15780993

[44] Nakajima C Uekusa Y Iwasaki M Yamaguchi N Mukai T Gao P. A role of interferon-gamma (IFN-gamma) in tumor immunity: T cells with the capacity to reject tumor cells are generated but fail to migrate to tumor sites in IFN-gamma-deficient mice. Cancer Res (2001) 61(8):3399–405. 11309299

[45] Uchida R Ashihara E Sato K Kimura S Kuroda J Takeuchi M. γδT cells kill myeloma cells by sensing mevalonate metabolites and ICAM-1 molecules on cell surface. Biochem Biophys Res Commun (2007) 354:613–8. doi: 10.1016/j.bbrc.2007.01.031 17250803

[46] Correia DV Fogli M Hudspeth K da Silva MG Mavilio D Silva-Santos B. Differentiation of human peripheral blood Vδ1+ T cells expressing the natural cytotoxicity receptor NKp30 for recognition of lymphoid leukemia cells. Blood (2011) 118:992–1001. doi: 10.1182/blood-2011-02-339135 21633088

[47] Wang H Henry O Distefano MD Wang Y-C Räikkönen J Mönkkönen J. Butyrophilin 3A1 plays an essential role in prenyl pyrophosphate stimulation of human Vγ2Vδ2 T cells. J.I (2013) 191:1029–42. doi: 10.4049/jimmunol.1300658 PMC3884521 23833237

[48] Girlanda S Fortis C Belloni D Ferrero E Ticozzi P Sciorati C. MICA expressed by multiple myeloma and monoclonal gammopathy of undetermined significance plasma cells costimulates pamidronate-activated γδ lymphocytes. Cancer Res (2005) 65:7502–8. doi: 10.1158/0008-5472.CAN-05-0731 16103105

[49] Rincon-Orozco B Kunzmann V Wrobel P Kabelitz D Steinle A Herrmann T. Activation of Vγ9Vδ2 T cells by NKG2D. J Immunol (2005) 175:2144–51. doi: 10.4049/jimmunol.175.4.2144 16081780

[50] Bürk MR Mori L de Libero G. Human Vγ9-Vδ2 cells are stimulated in a crossreactive fashion by a variety of phosphorylated metabolites. Eur J Immunol (1995) 25:2052–8. doi: 10.1002/eji.1830250737 7621879

[51] Kistowska M Rossy E Sansano S Gober H-J Landmann R Mori L. Dysregulation of the host mevalonate pathway during early bacterial infection activates human TCR γδ cells. Eur J Immunol (2008) 38:2200–9. doi: 10.1002/eji.200838366 18624305

[52] Fisher JP Flutter B Wesemann F Frosch J Rossig C Gustafsson K. Effective combination treatment of GD2-expressing neuroblastoma and ewing’s sarcoma using anti-GD2 ch14.18/CHO antibody with Vgamma9Vdelta2+ gammadeltaT cells. Oncoimmunology (2016) 5:e1025194. doi: 10.1080/2162402X.2015.1025194 26942051 PMC4760299

[53] Brandes M Willimann K Moser B. Professional Antigen-Presentation Function by Human γδ T Cells. Science (2005) 309:264–268. doi: 10.1126/science.1110267 15933162

[54] Himoudi N Morgenstern DA Yan M Vernay B Saraiva L Wu Y. Human γδ T Lymphocytes Are Licensed for Professional Antigen Presentation by Interaction with Opsonized Target Cells. J Immunol (2012) 188(4):1708–1716. doi: 10.4049/jimmunol.1102654 22250090

[55] Wu Y Wu W Wong WM Ward W Thrasher AJ Goldblatt D. Human γδ T Cells: A Lymphoid Lineage Cell Capable of Professional Phagocytosis. J Immunol (2009) 183(9):5622–5629. doi: 10.4049/jimmunol.0901772 19843947

[56] Davey MS Lin C-Y Roberts GW Heuston S Brown AC Chess JA. Human neutrophil clearance of bacterial pathogens triggers anti-microbial γδ T cell responses in early infection. PloS Pathog (2011) 7:e1002040. doi: 10.1371/journal.ppat.1002040 21589907 PMC3093373

[57] Barisa M Kramer AM Majani Y Moulding D Saraiva L Bajaj-Elliott M. E. coli promotes human Vgamma9Vdelta2 T cell transition from cytokine-producing bactericidal effectors to professional phagocytic killers in a TCR-dependent manner. Sci Rep (2017) 7:2805. doi: 10.1038/s41598-017-02886-8 28584241 PMC5459831

[58] Cui Y Kang L Cui L He W. Human γδ T cell recognition of lipid a is predominately presented by CD1b or CD1c on dendritic cells. Biol Direct (2009) 4:1–12. doi: 10.1186/1745-6150-4-47 19948070 PMC3224963

[59] Hui L Dai Y Guo Z Jiahui Z Zheng F Bian X. Immunoregulation effects of different γδT cells and toll-like receptor signaling pathways in neonatal necrotizing enterocolitis. Medicine (2017) 96:e6077. doi: 10.1097/MD.0000000000006077 28225489 PMC5569415

[60] Tisoncik JR Korth MJ Simmons CP Farrar J Martin TR Katze MG. Into the eye of the cytokine storm. Microbiol Mol Biol Rev (2012) 76:16–32. doi: 10.1128/MMBR.05015-11 22390970 PMC3294426

[61] Saraiva M Vieira P O’Garra A. Biology and therapeutic potential of interleukin-10. J Exp Med (2020) 217:e20190418. doi: 10.1084/jem.20190418 31611251 PMC7037253

[62] Liang X Cheng H Liu C Liu G. Antigen self-presenting nanovaccine for cancer immunotherapy. Sci Bull (2022) 67:1611–3. doi: 10.1016/j.scib.2022.07.018 36546034

[63] Li Y Zhao R Cheng K Zhang K Wang Y Zhang Y. Bacterial outer membrane vesicles presenting programmed death 1 for improved cancer immunotherapy *via* immune activation and checkpoint inhibition. ACS Nano (2020) 14:16698–711. doi: 10.1021/acsnano.0c03776 33232124

[64] Chen H Zhang P Shi Y Liu C Zhou Q Zeng Y. Functional nanovesicles displaying anti-PD-L1 antibodies for programmed photoimmunotherapy. J Nanobiotechnol (2022) 20:61. doi: 10.1186/s12951-022-01266-3 PMC8811970 35109867

