# Supplementary material for: Bacterial outer-membrane vesicles promote Vγ9Vδ2 T cell oncolytic activity
Source: Front Immunol. 2023 Jul 17;14:1198996. doi: 10.3389/fimmu.2023.1198996 (PMC10388717; doi:10.3389/fimmu.2023.1198996)
Supplement: Supplementary file 1 [file Table_1.docx]

|  | | | | |
| --- | --- | --- | --- | --- |
| **Experiment** | **Target** | **Antibody** | **Clone** | **Source** |
| PBMC stimulation with *E. coli* MG1655 Δ*pal* Δ*lpxM* OMVs | CD3 | CD3-BUV395 | SK7 | BD Biosciences |
|  | αβTCR | αβTCR-PE | T10B9 | BD Biosciences |
|  | CD69 | CD69-AF488 | FN50 | Biolegend |
|  | CD86 | CD86-Bv421 | IT2.2 | Biolegend |
|  | CD107a | CD107a-APC | H4A3 | BD Biosciences |
| Isotype controls | - | BUV395 Ms IgG1κ | X40 | BD Biosciences |
|  | - | PE Ms IgMκ | MM-30 | BD Biosciences |
|  | - | AF488 Ms IgG1κ | MOPC-21 | Biolegend |
|  | - | Bv421 Ms IgG2bκ | MPC-11 | Biolegend |
|  | - | APC Mouse BALB/c IgG1κ | MOPC-21 | BD Biosciences |
| γδ T cell activation with *E. coli* MG1655 Δ*pal* Δ*lpxM* OMVs | CD3 | CD3-BUV395 | SK7 | BD Biosciences |
|  | αβTCR | αβTCR-PE | T10B9 | BD Biosciences |
|  | Vδ1 | Vδ1-PE Cyan | TS8.2 | Life Technologies |
|  | Vδ2 | Vδ2-Bv711 | B6 | Biolegend |
|  | CD56 | CD56-Bv786 | NCAM16.2 | BD Biosciences |
| γδ T cell isolation purity/γδ T cell-mediated Nalm6 cell killing | CD3 | CD3-BUV395 | SK7 | BD Biosciences |
|  | αβTCR | αβTCR-PE | T10B9 | BD Biosciences |
