# Supplementary material for: Bacterial outer-membrane vesicles promote Vγ9Vδ2 T cell oncolytic activity
Source: Front Immunol. 2023 Jul 17;14:1198996. doi: 10.3389/fimmu.2023.1198996 (PMC10388717; doi:10.3389/fimmu.2023.1198996)
Supplement: Supplementary file 2 [file DataSheet_1.docx]

Supplementary Material

**1. Supplementary Figures and Tables**

**1.1 Supplementary Tables**

**Supplementary Table 1 –** Antibodies used for flow cytometry.

**1. 2 Supplementary Figures**

**Supplementary Figure 1 –** Comparison of γδ T cell gating methods after expansion from PBMC of three healthy donors (HD1, HD2 and HD3) (A). Purity of isolated γδ T cells as a percentage of the total cell population after expansion with either zoledronate or *E. coli* MG1655 Δ*pal* Δ*lpxM* OMVs (OMV) (B). Data are presented as the mean ± SD (n=3), ns=non-significant, analysed by paired Welsch’s t-test (A).

**Supplementary Figure 2 –** Activation marker expression on ⍺β T cells (A & B), γδ T cells (C & D) and NK cells (E & F) from two healthy donors, five days after stimulation of PBMC with PBS (Mock), and *E. coli* MG1655 Δ*pal* Δ*lpxM* OMVs at 1x10^4^ vesicles per cell (10000:1), and 1x10^3^ vesicles per cell (1000:1). Expression measured using median fluorescence intensity (MFI). Data are presented as the mean ± SD from representative experiments (n=3). ****P<0.0001, ***P<0.001, **P<0.01, *P < 0.05, analysed by one-way ANOVA with Tukey’s post-test.

**Supplementary Figure 3 –** Flow cytometry plots reflecting activation marker expression on ⍺β T cells, γδ T cells and NK cells, 5 days after stimulation of PBMC with PBS (dashed black line), *E. coli* MG1655 Δ*pal* Δ*lpxM* OMVs at 1x10^4^ vesicles per cell (10000:1) (solid black line), and 1x10^3^ vesicles per cell (1000:1) (solid blue line). Isotype control is represented by the shaded grey area. Data are presented as representative plots from one donor.

**Supplementary Figure 4 –** Activation marker expression on ⍺β T cells (A, D, G), γδ T cells (B, E, H) and NK cells (C, F, I) from three healthy donors, five days after stimulation of PBMC with PBS (Mock), and *E. coli* MG1655 Δ*pal* Δ*lpxM* OMVs at 1x10^4^ vesicles per cell (10000:1), and 1x10^3^ vesicles per cell (1000:1). Expression measured as a proportion of cells positive for each marker. Data are presented as the mean ± SD from representative experiments (n=3). ****P<0.0001, ***P<0.001, **P<0.01, *P < 0.05, analysed by one-way ANOVA with Tukey’s post-test.

**Supplementary Figure 5 –** Flow cytometry plots reflecting the population of ⍺β T cells, γδ T cells and NK cells expressing activation markers, 5 days after stimulation of PBMC with PBS (Mock), *E. coli* MG1655 Δ*pal* Δ*lpxM* OMVs at 1x10^4^ vesicles per cell (1000:1), and 1x10^3^ vesicles per cell (10000:1). Isotype controls used to determine gating. Data are presented as representative plots from one donor.

**Supplementary Figure 6 –** Release of IFN-γ (A & B) and granzyme B (C & D) by PBMC from two healthy donors, five days after stimulation with *E. coli* MG1655 Δ*pal* Δ*lpxM* OMVs (OMV) at 1x10^4^ vesicles per cell, or PBS (Mock). Data are presented as the mean ± SD from representative experiments (n=3). ****P<0.0001, **P<0.01, *P < 0.05, analysed by unpaired Welsch’s t-test.

**Supplementary Figure 7 –** Total cell number within the PBMC population before stimulation (Pre-stim), and after a 10-day expansion period stimulated with IL-2 alone (IL-2), OMVs with IL-2 (OMV), and zoledronate with IL-2 (Zol). Data are presented as the mean ± SD (n=3). ns=non-significant, analysed by one-way ANOVA with Tukey’s post-test.

**Supplementary Figure 8 –** Flow cytometry plots demonstrating relative proportion of cytotoxic lymphocytes within the PBMC population before stimulation (CTRL), and after a 10 day expansion period stimulated with IL-2 alone (IL-2), *E. coli* MG1655 Δ*pal* Δ*lpxM* OMVs with IL-2 (OMV), and zoledronate with IL-2 (Zol). Data are presented as representative plots from one donor.
