# Supplementary figures and images for: Bacterial outer-membrane vesicles promote Vγ9Vδ2 T cell oncolytic activity
Source: Front Immunol. 2023 Jul 17;14:1198996. doi: 10.3389/fimmu.2023.1198996 (PMC10388717; doi:10.3389/fimmu.2023.1198996)

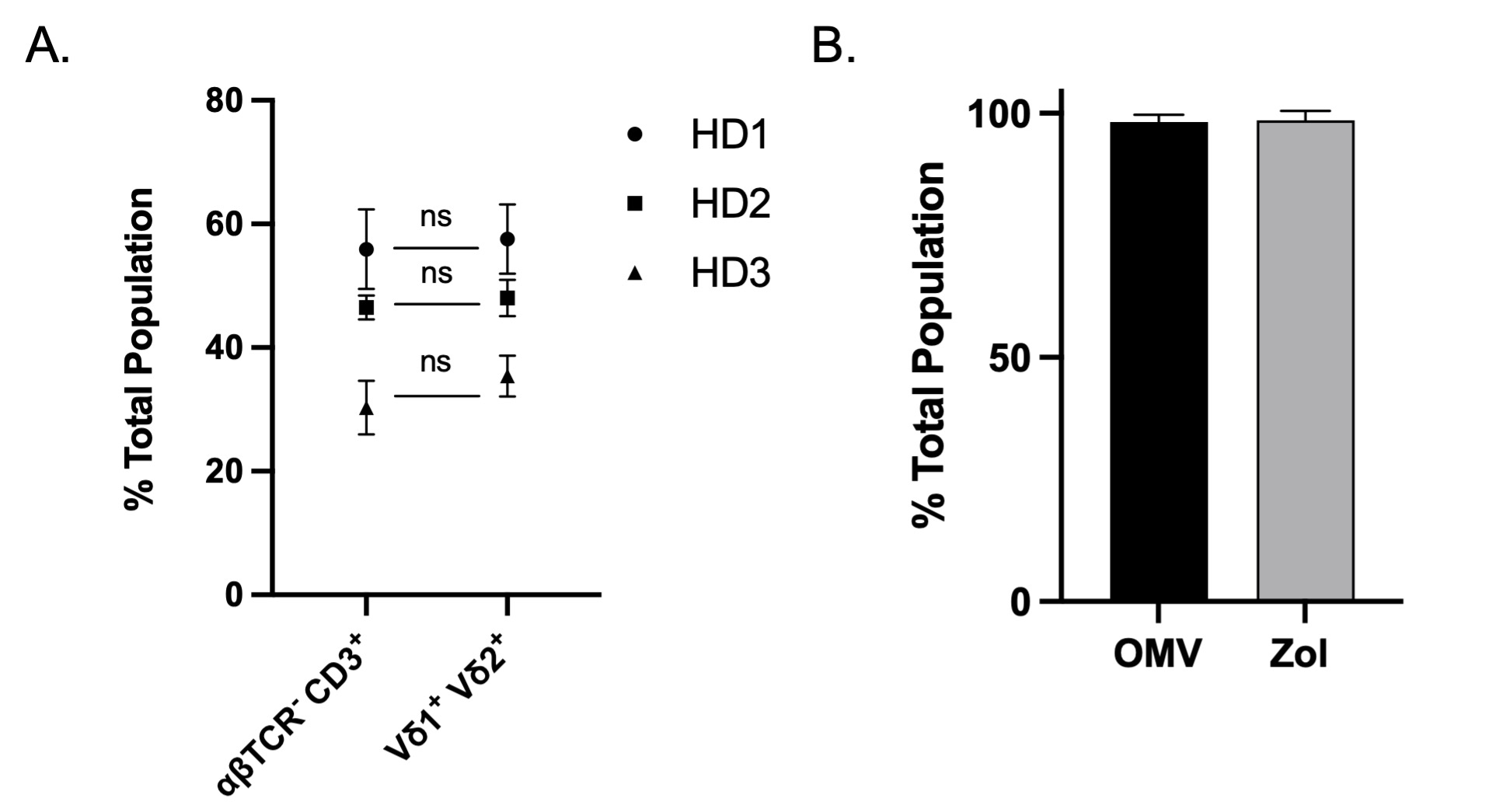

Supplement: Supplementary file 3 [file Image_1.jpeg]

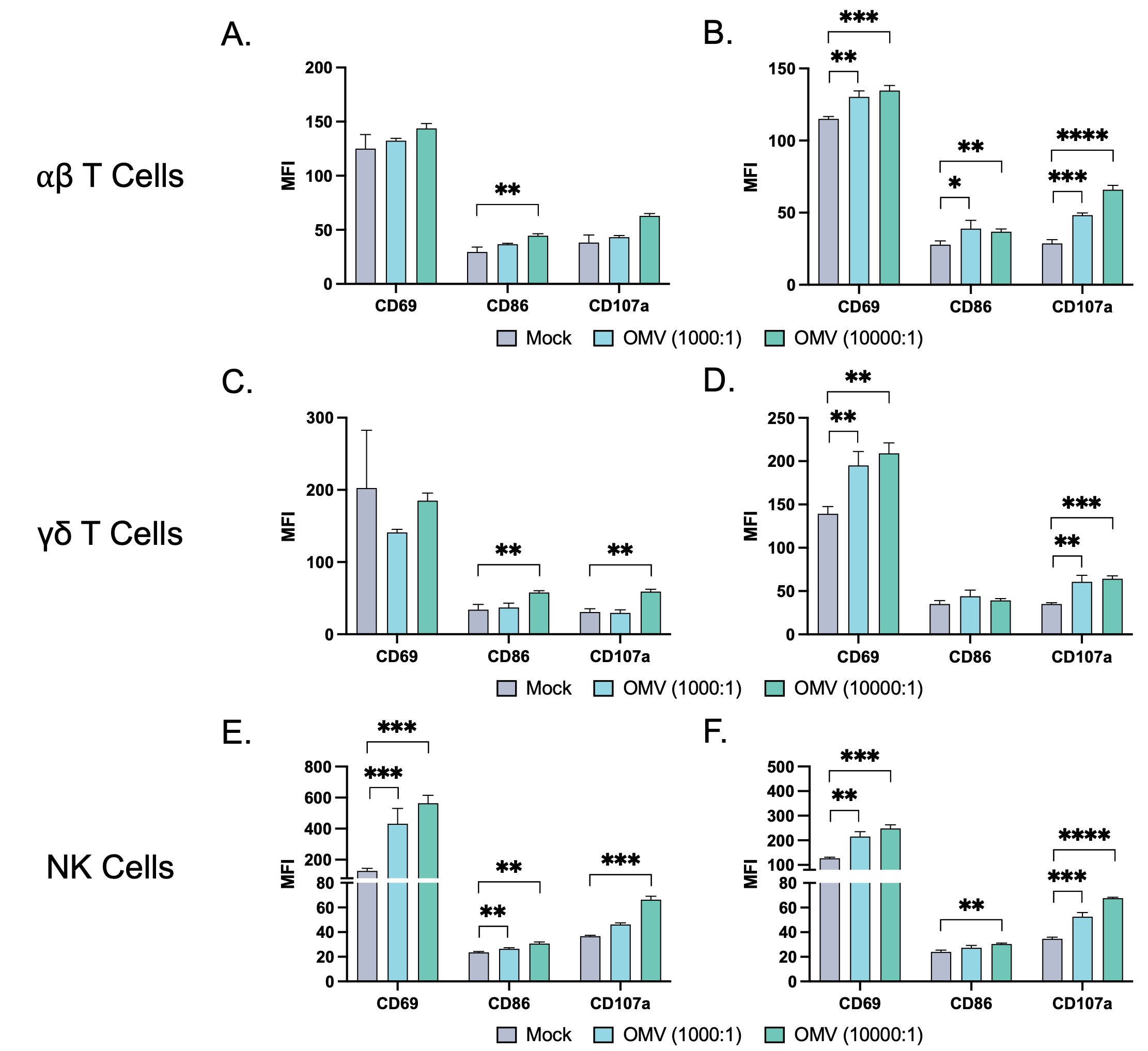

Supplement: Supplementary file 4 [file Image_2.jpeg]

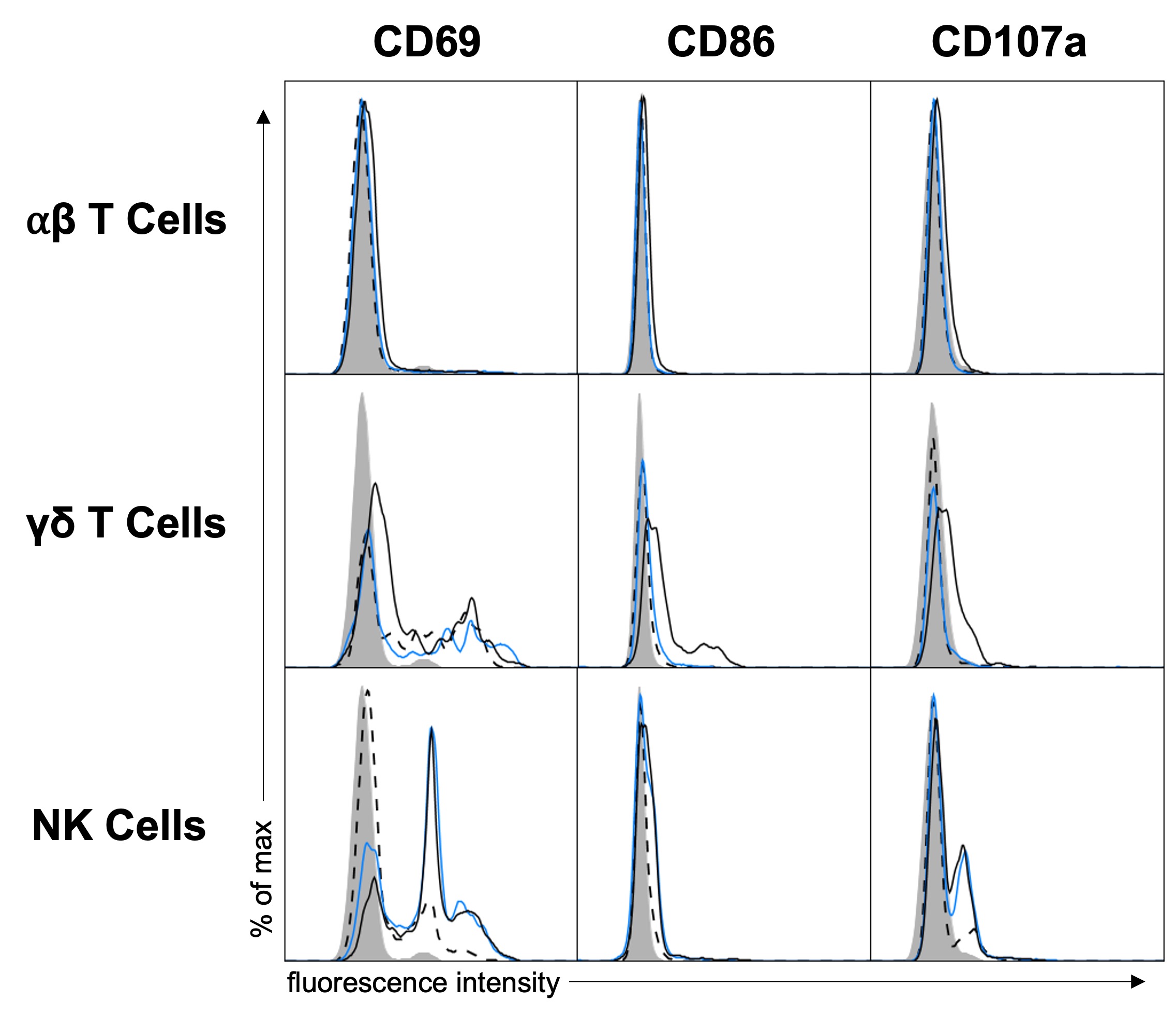

Supplement: Supplementary file 5 [file Image_3.jpeg]

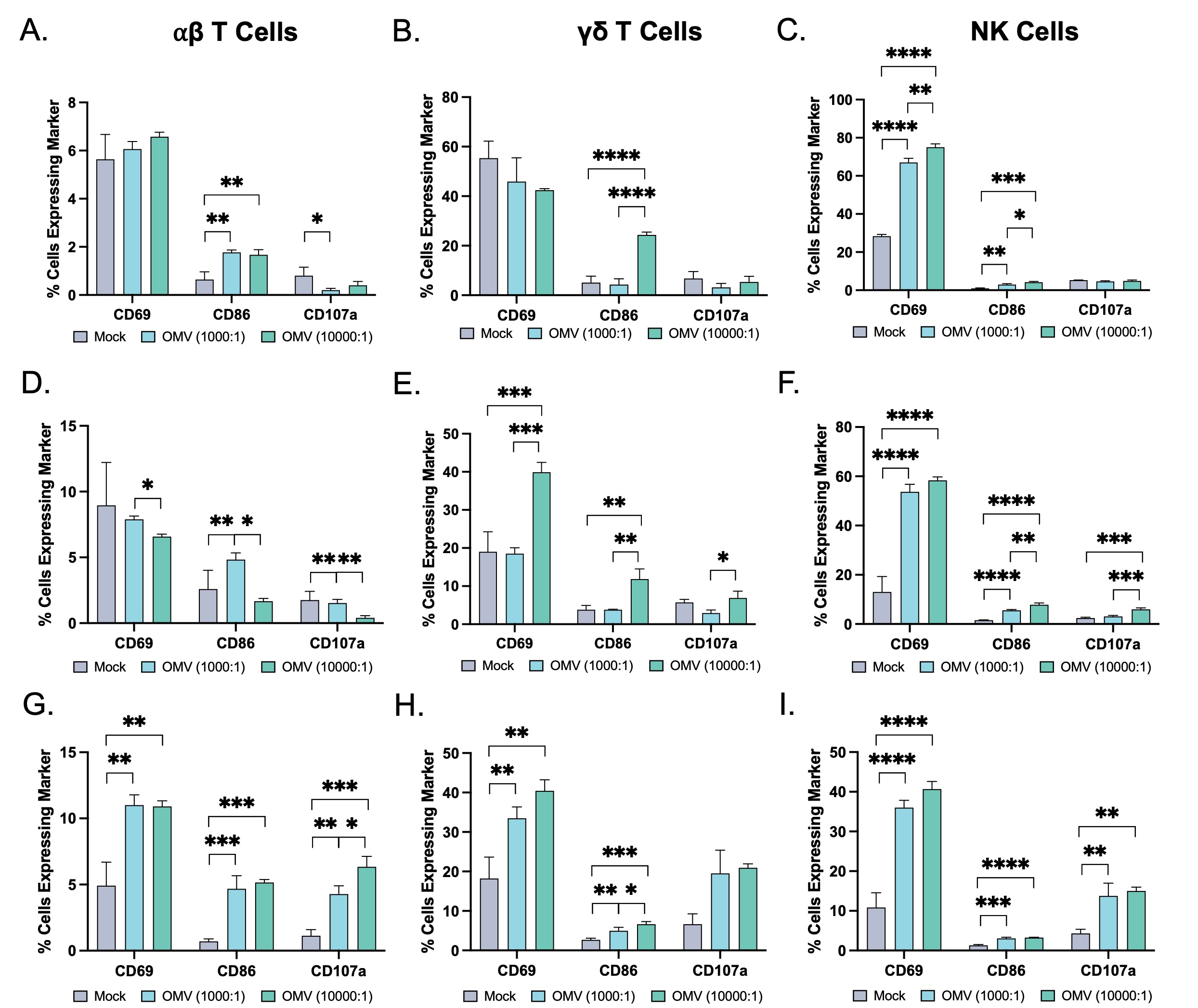

Supplement: Supplementary file 6 [file Image_4.jpeg]

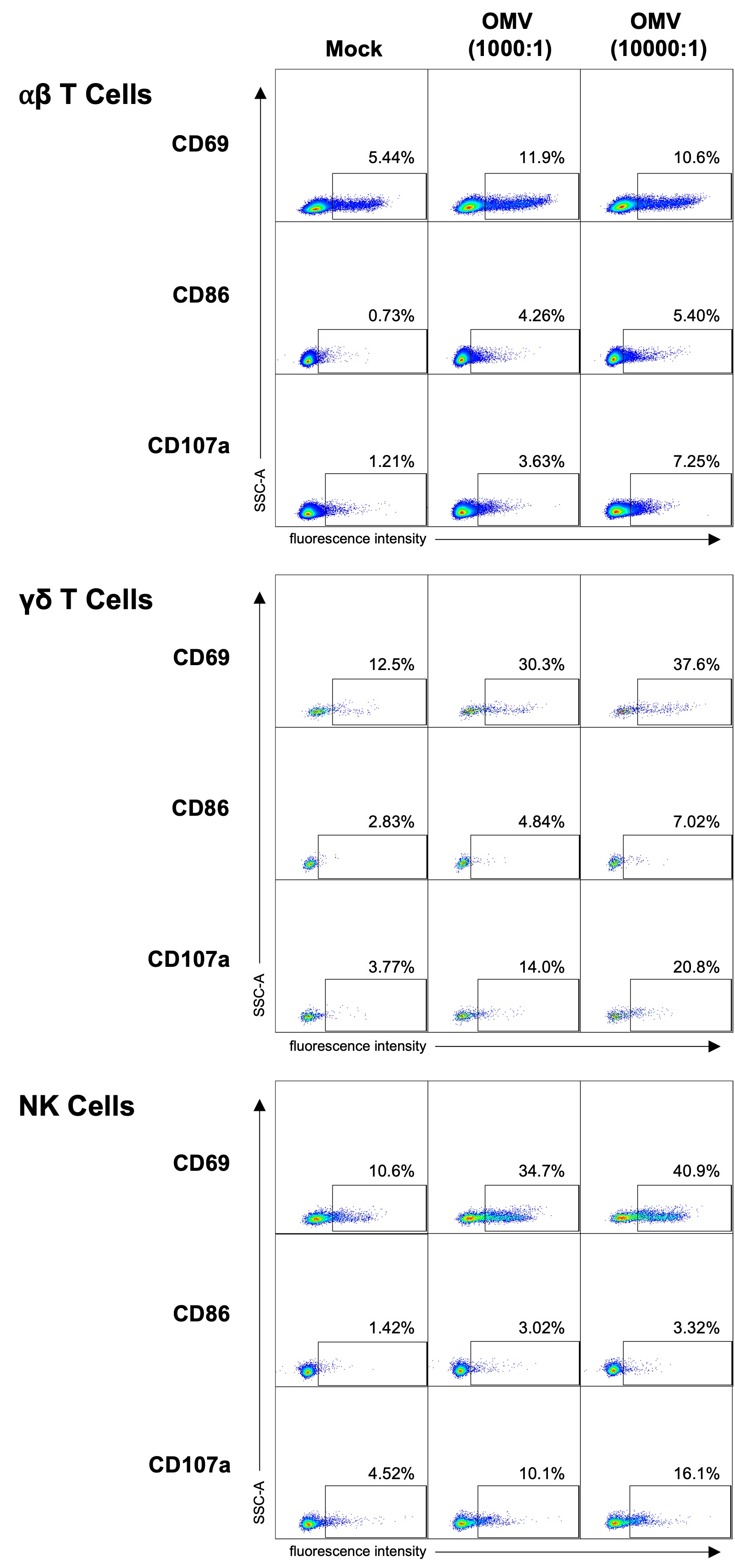

Supplement: Supplementary file 7 [file Image_5.jpeg]

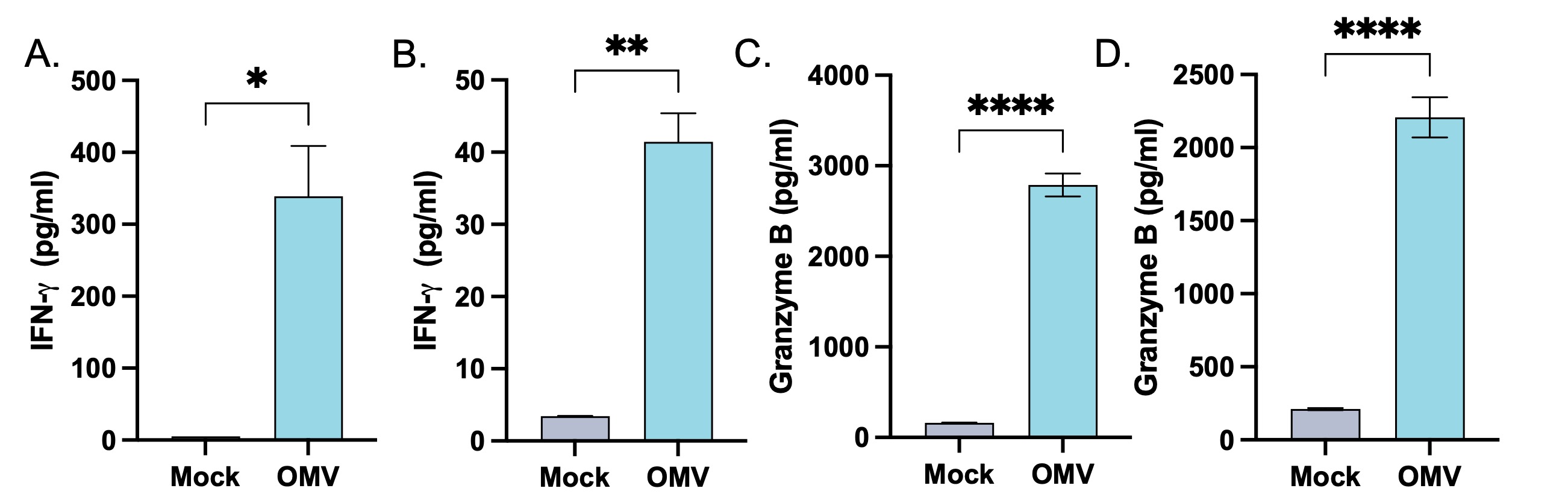

Supplement: Supplementary file 8 [file Image_6.jpeg]

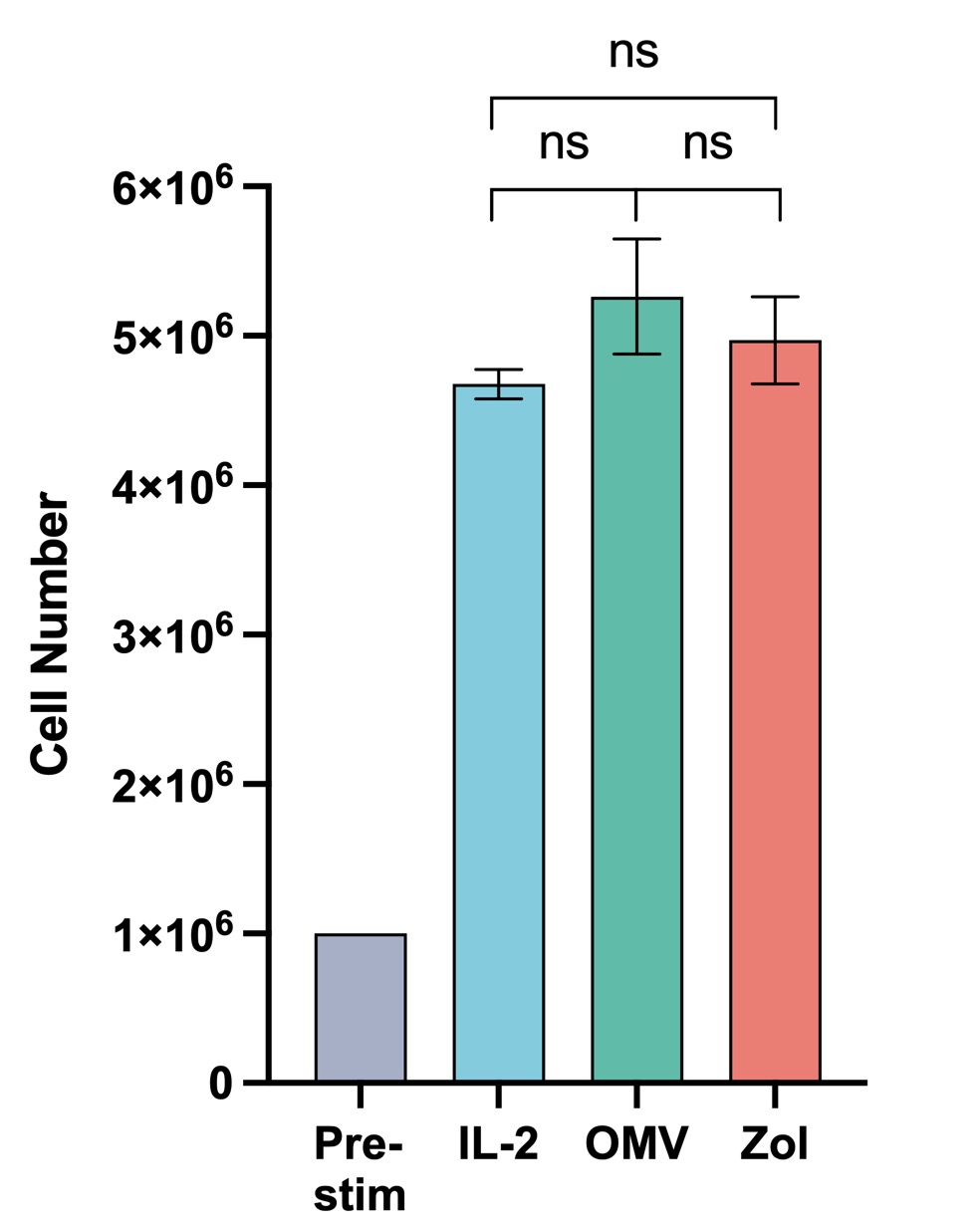

Supplement: Supplementary file 9 [file Image_7.jpeg]

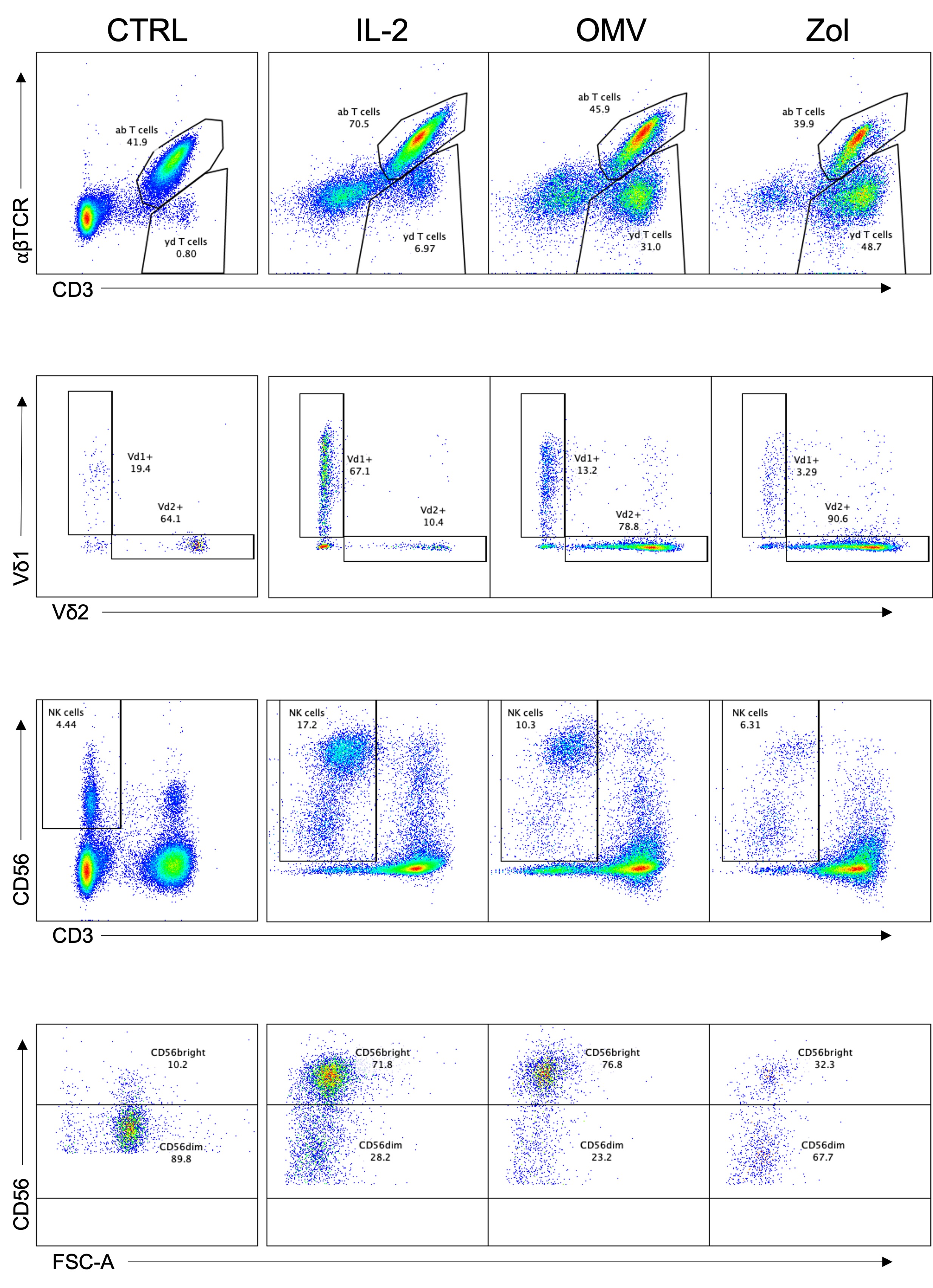

Supplement: Supplementary file 10 [file Image_8.jpeg]
